# Preparation, Characterization and Application of the Delivery System for Food Products

**DOI:** 10.3390/foods12234187

**Published:** 2023-11-21

**Authors:** Shuai Chen, Qing Guo

**Affiliations:** 1 School of Public Health, Wuhan University, Wuhan 430071, China; 2 School of Food Science and Technology, Tianjin University of Science & Technology, Tianjin 300457, China; qingguo@tust.edu.cn; 3 State Key Laboratory of Food Nutrition and Safety, Tianjin University of Science & Technology, Tianjin 300457, China

## 1. Introduction

In the dynamic and evolving landscape of food science and technology, the quest to develop innovative and effective delivery systems for bioactive compounds remains a focal point of research and development [1]. This pursuit is fueled by the ever-increasing demand for functional foods that offer not only enhanced nutritional value, but also targeted and controlled release of bioactive components [2], thereby imparting health benefits to consumers [3]. The Special Issue of the journal *Foods* represents a significant milestone in the ongoing exploration of delivery systems designed to revolutionize the way we consume and benefit from bioactive compounds.

## 2. Delivery Systems for Food Products

Bioactive compounds, such as polysaccharides, peptides, polyphenols, alkaloids, sterols, saponins, etc., have gained recognition for their potential to promote human health and well-being [4]. However, realizing the full therapeutic potential of these compounds often requires overcoming several challenges, including their stability during food processing and storage [5], controlled release in the gastrointestinal tract, and interaction with the complex milieu of the intestinal microbiota [6].

## 3. Controlled Release of Bioactive Compounds

Controlled release technologies have emerged as a key focus area within food science [7]. The ability to modulate the release of bioactive compounds in the digestive system not only enhances their bioavailability, but also ensures that they reach their intended target sites, where their effects are most pronounced [8,9]. In this Special Issue, we delve into the latest advancements in controlled release mechanisms, from encapsulation techniques to nanostructured delivery systems. We explore how these innovative approaches can safeguard bioactive compounds from degradation, improve their solubility, and enable their sustained release, ultimately enhancing their efficacy.

## 4. Enhancing Stability and Bioavailability

Stability and bioavailability are critical factors governing the efficacy of bioactive compounds in functional foods [10,11]. The articles in this Special Issue explore various strategies for enhancing the stability of these compounds during food processing and storage. Furthermore, they investigate how delivery systems can facilitate the release of bioactives at specific points along the digestive tract, optimizing their absorption and utilization by the body.

## 5. Resistant Starch: A Resilient Carrier

Resistant starch, a unique dietary fiber, has garnered attention for its potential as a carrier for bioactive compounds [12]. Its resistance to digestion in the small intestine allows it to serve as a selective delivery vehicle for bioactives to the colon, where they can interact with the diverse and influential community of intestinal flora. This Special Issue also explores the utilization of resistant starch as a carrier system, shedding light on its potential to modulate gut health and microbiota composition.

## 6. Gut Health

The impact of bioactive compounds on gut health is a burgeoning area of research, with implications far beyond digestion [13]. This Special Issue also includes articles that provide insights into how bioactive delivery systems can influence the gut microbiota, potentially leading to improved metabolic outcomes, enhanced immune function, and reduced risk of chronic diseases.

## 7. Functional Food: From Concept to Reality

Ultimately, the culmination of research relating delivery systems for bioactive compounds is the creation of functional foods that offer tangible health benefits [14,15]. This Special Issue bridges the gap between scientific inquiry and practical application, with a focus on translating research findings into food product development. From fortified beverages to novel snacks, the potential for functional foods is vast, and this Special Issue offers a glimpse into their exciting future.

## 8. Conclusions

As we embark on this journey through the pages of the Special Issue, we invite readers to explore the cutting-edge research, innovative technologies, and transformative insights that promise to shape the future of food science. This collection of articles represents a collaborative effort by researchers, scientists, and industry experts dedicated to harnessing the power of delivery systems to unlock the full potential of bioactive compounds in food products. Together, we embark on a path toward healthier, more nutritious, and more enjoyable foods that have the potential to revolutionize our approach to nutrition and well-being.
